# Immunogenicity and protective potential of chimeric virus-like particles containing SARS-CoV-2 spike and H5N1 matrix 1 proteins

**DOI:** 10.3389/fcimb.2022.967493

**Published:** 2022-07-18

**Authors:** Jing Chen, Wang Xu, Letian Li, Lichao Yi, Yuhang Jiang, Pengfei Hao, Zhiqiang Xu, Wancheng Zou, Peiheng Li, Zihan Gao, Mingyao Tian, Ningyi Jin, Linzhu Ren, Chang Li

**Affiliations:** ^1^ College of Veterinary medicine, Key Lab for Zoonoses Research, Ministry of Education, Jilin University, Changchun, China; ^2^ Research Unit of Key Technologies for Prevention and Control of Virus Zoonoses, Chinese Academy of Medical Sciences, Changchun Veterinary Research Institute, Chinese Academy of Agricultural Sciences, Changchun, China; ^3^ College of Animal Sciences, Key Lab for Zoonoses Research, Ministry of Education, Jilin University, Changchun, China

**Keywords:** severe acute respiratory syndrome coronavirus type 2 (SARS-CoV-2), Coronavirus Disease 2019 (COVID-19), virus-like particle (VLP), spike, chimeric

## Abstract

Coronavirus Disease 2019 (COVID-19), caused by severe acute respiratory syndrome coronavirus type 2 (SARS-CoV-2), has posed a constant threat to human beings and the world economy for more than two years. Vaccination is the first choice to control and prevent the pandemic. However, an effective SARS-CoV-2 vaccine against the virus infection is still needed. This study designed and prepared four kinds of virus-like particles (VLPs) using an insect expression system. Two constructs encoded wild-type SARS-CoV-2 spike (S) fused with or without H5N1 matrix 1 (M1) (S and SM). The other two constructs contained a codon-optimized spike gene and/or M1 gene (mS and mSM) based on protein expression, stability, and ADE avoidance. The results showed that the VLP-based vaccine could induce high SARS-CoV-2 specific antibodies in mice, including specific IgG, IgG1, and IgG2a. Moreover, the mSM group has the most robust ability to stimulate humoral immunity and cellular immunity than the other VLPs, suggesting the mSM is the best immunogen. Further studies showed that the mSM combined with Al/CpG adjuvant could stimulate animals to produce sustained high-level antibodies and establish an effective protective barrier to protect mice from challenges with mouse-adapted strain. The vaccine based on mSM and Al/CpG adjuvant is a promising candidate vaccine to prevent the COVID-19 pandemic.

## Introduction

Coronavirus can infect many kinds of animals, and it can also cause mild to severe respiratory tract infections in humans. Since it was first reported in China in December 2019, severe acute respiratory syndrome coronavirus type 2 (SARS-CoV-2), as well as two zoonotic highly pathogenic coronaviruses SARS-CoV and Middle East respiratory syndrome coronavirus (MERS-CoV), which appeared in 2002 and 2012 respectively, have made coronavirus infection a new and vital public health problem in the 21st century (Cui et al., 2019).

Significantly, the continuous emergence of the SARS-CoV-2 variants of concern (VOCs), such as Delta, Omicron, and their derivates, have posed a constant threat to human beings and the world economy (Hui et al., 2020; Li et al., 2021a). Although the vaccine is the best choice to prevent virus infection, the main target of current vaccines is viral Spike protein, which may escape the recognition of the vaccine through mutation over time, resulting in the decline of the protection rate of vaccines (Callaway, 2021a; Flanagan et al., 2021; Mccallum et al., 2021; Murano et al., 2021; Pulliam et al., 2022). As reported, mutations in the Spike of variants Kappa and Delta eliminated the recognition of several monoclonal antibodies by changing key antigenic sites, including remodeling the N-terminal domain of the Spike and acquiring an N-linked glycan in the receptor-binding domain (RBD), with a markedly reduced affinity between the Spike and receptor Angiotensin-converting enzyme 2 (ACE2) (Wrapp et al., 2020; Flanagan et al., 2021; Mccallum et al., 2021; Murano et al., 2021; Nabel et al., 2022). These results were similar to the antigenic evolution of the viral Spike in coronavirus 229E, especially in the RBD (Eguia et al., 2021). Furthermore, the currently dominant VOC Omicron harbors more mutations than other variants reported previously, suggesting that SARS-CoV-2 variants further adapt to the human body through continuous mutation, especially in immune-compromised individuals during chronic infection (Callaway, 2021b; Li et al., 2021a; Pulliam et al., 2022). A phylogenetic tree based on the mutations in the S1 subunit of viral Spike showed that the Omicron is far from other variants, indicating that Omicron evolved in parallel with other variants (Kupferschmidt, 2021). These results suggest that the Omicron may escape the vaccine and has enhanced infectivity and/or pathogenicity, proved in the previously reported VOCs. Moreover, although currently available vaccines can induce SARS-CoV-2-specific immune responses, SARS-CoV-2 variants can still bypass the pre-existing immunity and spread to both immature and seroconverted individuals (Horiuchi et al., 2021; Ying et al., 2022), which indicates that virus transmission still occurs in people, even in the vaccinated people and animal reservoirs (Kupferschmidt, 2021). It is one of the reasons why many countries urgently have begun to administrate the third and/or fourth dose of the SARS-CoV-2 vaccine. Therefore, a more efficient single-dose or two-doses SARS-CoV-2 vaccine against the emerging VOCs is still needed.

To date, more than 20 vaccines have been authorized for emergency use, and more than 11,242,252,352 vaccine doses have been administered on 4 April 2022. However, many different platforms of the SARS-CoV-2 vaccine are still under development, based on recombinant vectors, DNA, mRNA, inactivated viruses, attenuated viruses, and protein subunits, etc. (Gao et al., 2020; Smith et al., 2020; Wang et al., 2020a; Zhu et al., 2020; Flanagan et al., 2021; Li et al., 2021b). In addition, evaluation of possible targets and pan-coronavirus antiviral strategies for emerging or re-emerging coronaviruses is also in progress (Li et al., 2021b). Among these strategies, the vaccine based on the virus-like particle (VLP) has attracted more and more attention.

VLP comprises a virus capsid without a virus genome and can be used as multifunctional, safe, and highly immunogenic vaccines (Roy and Noad, 2008; Liu et al., 2011; Lopez-Macias et al., 2011; Matsuda et al., 2020; Won and Lee, 2020; Prates-Syed et al., 2021; Tariq et al., 2021; Kim et al., 2022). Moreover, the repeated antigen pattern on the surface of VLP makes it easier to be recognized by antigen-presenting cells than subunit vaccines to induce more robust and broader humoral and cellular immune responses (Bright et al., 2007; Bright et al., 2008; Song et al., 2010). Therefore, the VLP-based vaccine may be safer than inactivated or attenuated virus vaccine and more immunogenic than subunit or DNA vaccines. Besides, there were reported that Matrix 1 (M1) from avian influenza A H5N1 can promote the efficient assembly of influenza VLP (Bright et al., 2008; Song et al., 2010). Furthermore, a previous study also indicated that the chimeric VLPs containing the SARS-CoV-1 Spike (with a replacement of the TM/CT by the corresponding sequence of influenza virus HA) and the influenza M1 could be highly expressed in the baculovirus insect cell expression system (Liu et al., 2011). Moreover, influenza VLPs expressing the SARS-CoV-2 Spike can induce high levels of humoral immune responses against SARS-CoV-2, whereas neutralizing activities of the antibody were not strong enough to completely inhibit receptor-ligand binding of the SARS-CoV-2 (Chu et al., 2021b). Therefore, in this study, the *Spike* (S) gene of SARS-CoV-2 was optimized, and the chimeric VLPs based on the optimized Spike of SARS-CoV-2 and H5N1 M1 protein were constructed by a baculovirus system. In addition, the immunogenicity and protective effect of the VLP-based vaccine were also verified.

## Materials and methods

### Plasmid construction

**Figure 1:**
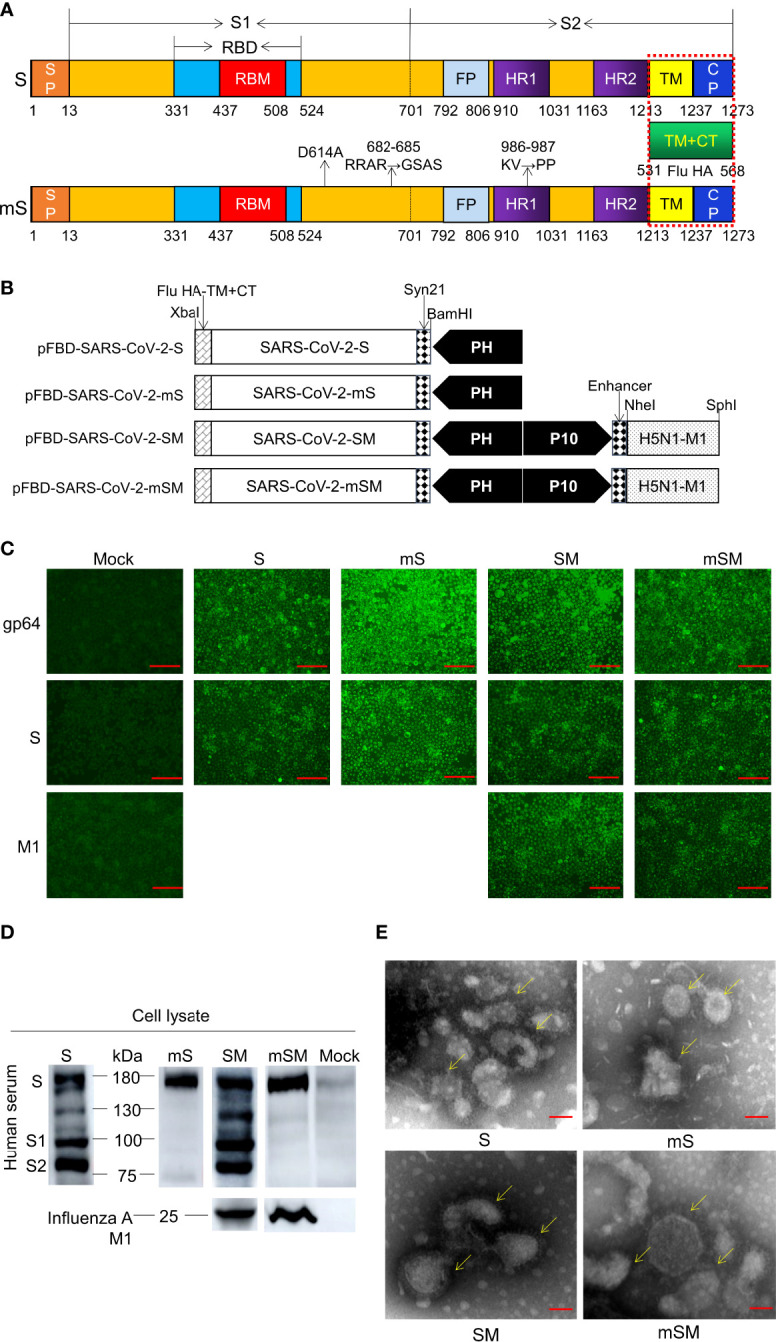
Generation of SARS-CoV-2 VLPs. **(A)** Schematic of SARS-CoV-2 S/M1 and its mutation. **(B)** Schematics of the four recombinant bacmid rFBD-SARS-CoV-2. **(C, D)** Expression of exogenous genes by recombinant baculoviruses identified by IFA **(C)** and Western blot **(D)**. The scale bar corresponds to 150 μm. Convalescent serum of COVID-19 patient or Influenza A M1 as the primary antibody, and HRP-labeled Goat Anti-Mouse IgG (H+L) as the secondary antibody. Mock, wild baculoviruses infected cell. Unprocessed original images can be found in Supplemental Figure S6. **(E)** Transmission electron micrograph of negatively stained SARS-CoV-2 VLPs. The scale bar corresponds to 50 nm.

The *spike* (S) gene of SARS-CoV-2 was designed according to the sequence of SARS-CoV-2/Wuhan-Hu-1 (GenBank ID: MN908947) (Yang et al., 2004) by adding the *Bam*HI and *Xba*I at the 5’ and 3’ terminal of the gene, respectively. Then, a Syn21 sequence (AACTTAAAAAAAAAAATCAAA) (Liu et al., 2015) was also inserted before the start codon (ATG) of the *spike* gene to enhance transcription. Furthermore, the transmembrane and carboxyl terminus (TM/CT) of S was replaced by the corresponding transmembrane sequence of H5N1 hemagglutinin (HA) (aa 531-568, 38aa, A/Indonesia/5/2005 M1) (Liu et al., 2011). The resulting sequence was named the *S* gene (**Figure 1A**).

As reported, three sites of the Spike, including D614, RRAR (682-685), and KV986-987, are crucial for the virus infection. The mutation of D614 to A614 may avoid Antibody-dependent enhancement (ADE) (Wang et al., 2016). The mutation of RRAR (682-685) to GSAS can inhibit the recognition and cleavage by furin (Kalnin et al., 2021). Finally, the mutation of KV986-987 to PP986-987 can enhance immunogenicity (Kirchdoerfer et al., 2018; Dagotto et al., 2020; Wrapp et al., 2020). Therefore, these sites were replaced by the corresponding sequence, respectively, and the optimized sequence was designated the *mS* gene (**Figure 1A**). The *S* gene and *mS* gene were synthesized according to the codon usage preference of insect cells and linked downstream of promoter PH of pFastBac™ Dual donor plasmid (Invitrogen, USA) according to the protocol described previously (Xu et al., 2021). The resulting shuttle plasmids were designated as pFBD-SARS-CoV-2-S and pFBD-SARS-CoV-2-mS, respectively (**Figure 1B**).

Moreover, to improve the stability of the VLP (Liu et al., 2011), the expression frame of the H5N1 *M1* gene (A/Indonesia/5/2005 M1, 252aa) was added to the downstream promoter P10 of pFastBac™ Dual donor plasmid (Invitrogen, USA), generating two shuttle plasmids pFBD-SARS-CoV-2-SM and pFBD-SARS-CoV-2-mSM, respectively (**Figure 1B**).

### Baculovirus rescue and VLP preparation

To generate recombinant bacmid, the donor plasmids pFBD-SARS-CoV-2-S, pFBD-SARS-CoV-2-mS, pFBD-SARS-CoV-2-SM, and pFBD-SARS-CoV-2-mSM were transformed into Competent DH10Bac™ *E. coli* cells, followed by the antibiotic selection. The positive *E. coli* colonies containing recombinant bacmids rBD-S, rBD-mS, rBD-SM, and rBD-mSM was identified by PCR. In addition, the recombinant bacmids were verified by PCR (**Table 1**).

**Table 1:** Primers and probes used in this study.

Primers		Sequence (5’-3’)	Length (bp)	Gene Name
1	SF	AATGTTCGTGTTCTTGGTCTTGC	3800	S
	pUC/M13R	AGCGGATAACAATTTCACACAGG		
2	M1F	ATGAGCCTGTTGACCGAGGT	750	M1
	M1R	CTACTTGAAACGCTGCATC		
3	mSF	AATGTTCGTGTTCTTGGTCTTGC	3800	mS
	pUC/M13R	AGCGGATAACAATTTCACACAGG		
4	M1F	ATGAGCCTGTTGACCGAGGT	1500	M1
	pUC/M13F	CCCAGTCACGACGTTGTAAAACG		
5	SARS-CoV-2 NF	GGGGAACTTCTCCTGCTAGAAT	99	N
	SARS-CoV-2 NR	CAGACATTTTGCTCTCAAGCTG		
	SARS-CoV-2 NP	5’-FAM-TTGCTGCTGCTTGACAGATT-TAMRA-3’	Probe	
6	SARS-CoV-2 SgEF	CGATCTCTTGTAGATCTGTTCTC	171	E
	SARS-CoV-2 SgER	ATATTGCATTGCAGCAGTACGCACA		
	SARS-CoV-2 SgEP	5’-FAM-ACACTAGCCATCCTTACTGCGCTTGC-TAMRA-3’	Probe	

Recombinant baculovirus was rescued according to the previously described protocol (Xu et al., 2021). Briefly, sf9 cells in a 6-well plate (1×10^6^cells/well) were transfected with the recombinant bacmids using Cellfectin^®^ II Reagent (Gibco, USA) according to the manufacturer’s instructions and cultured in Grace’s medium (Gibco, USA) for 5-7 days at 27°C until apparent cytopathic effect (CPE) appeared. Then, cells and supernatant were collected, centrifuged at 3000 rpm, 4°C for 5 min, and filtered with a 0.22 μm filter. Next, the pellets were resuspended in PBS and evaluated using indirect fluorescence assay (IFA), Western blot, and transmission electron microscopy (TEM). The recombinant baculoviruses were named rBDV-S, rBDV-mS, rBDV-SM, and rBDV-mSM. Afterward, the baculoviruses were blind passaged in Sf9 cells and stored at -80°C. Finally, the viral titer of the third passage was examined according to the protocol described by BacPAK Baculovirus Rapid Titer Kit (Clontech, USA).

To generate VLPs, sf9 cells in a shake flask (1×10^6^cells/well) were infected with the third passage recombinant baculoviruses (multiplicity of infection, MOI=1) for 72 h. Medium supernatant was collected and ultracentrifuged through a 20% sucrose cushion at 30,000rpm, 4°C for 2 h. The pellets were resuspended in PBS and further purified using a stepwise sucrose density gradient consisting of 2 mL of 10%, 30%, and 60% sucrose in PBS and centrifuged at 30,000 rpm and 4°C for 2 h. The white circular target protein in 10%-30% was collected and transferred to the centrifuge tube containing 5mL PBS and centrifugated at 30,000 rpm, 4°C for 2 h. The pellets were resuspended in PBS and evaluated using SDS-PAGE and Western blot. At the same time, the target protein was also examined by SARS-CoV-2 (2019-nCoV) Spike ELISA Kit (Sino Biological, China) according to the manufacturer’s instructions. The standard curve was drawn according to the mean absorbance of optical density 450 (OD 450), and the concentration of the target antigen was calculated based on the standard curve.

### Indirect fluorescence assay

Sf9 cells (5×10^5^ cells/well) cultured in a 6-well plate were infected with the recombinant baculoviruses (MOI=1) for 48 h at 27°C. The IFA was performed as described by Xu *et al.* (Xu et al., 2021). Briefly, cells were fixed in 4% paraformaldehyde for 10 min at room temperature and washed three times with PBS for 5 min each. Next, the cells were blocked with 1% skim milk at 37°C for 30 min. After that, the cells were incubated with 500 μL AcMNPV GP64 antibody (1:1000, Sino Biological, China), SARS-CoV-2 (2019-nCoV) Spike RBD Antibody (1:1000, Sino Biological, China), or Influenza A M1 (1:1000, GeneTex, USA) at 37°C for 120 min. Then, the cells were incubated with 500 μL FITC-labeled Goat Anti-Rabbit IgG (H+L) (1:2,000, Beyotime, China) at 37°C for 40 min in the darkroom. Finally, the cells were examined using an Eclipse TE2000-V (Nikon, Japan).

### Western Blot

Western blot was performed according to the protocol described by Xu et al. (Xu et al., 2021). Briefly, cell lysate or protein solution was loaded (1:5) and separated *via* 10% SDS-PAGE. Then, the protein was transferred onto the PVDF membrane, blocked with 5% skim milk for 1 h at room temperature, followed by incubating with the convalescent serum of the COVID-19 patient (1:1000) or Influenza A M1 (1:1000, GeneTex, USA) for 2 h at room temperature. After that, the membrane was incubated with HRP-labeled Goat Anti-Rabbit IgG (H+L) or HRP-labeled Goat Anti-Mouse IgG (H+L) (1:3000, Beyotime, China) for 1 h at room temperature. Subsequently, the band was developed using the GEGEGNOME XRQ enhanced chemiluminescence (ECL) (Thermo Fisher SCIENTIFIC, USA).

### Transmission electron microscopy

The sample was absorbed on the grid and stained with 1% Phosphotungstic Acid for 1-2 min. Then, TEM was performed to examine the VLPs using a JEM-1200EXII transmission electron microscope (JEOL USA, Peabody, MA, USA).

### Animal immunization and challenge

Specific pathogen-free (SPF, 6-8 weeks old) Balb/c mice and C57/BL/6N mice were purchased from Vital River Laboratory (Beijing, China). In addition, Humanized Balb/c mice containing human ACE2 (Balb/c-hACE2) were constructed by Cyagen (Suzhou, China). Mice were housed in SPF stainless steel with a constant atmosphere (22-25°C, 45-50% relative humidity), natural light cycle, and unlimited feeding and drinking. The virus infection experiment was conducted in the BSL-3 laboratory, Changchun Institute of Veterinary Medicine, Chinese Academy of Agricultural Sciences. Mice were monitored three times daily for changes in physical appearance and deaths (if any) and weighed every day during the experiment. In addition, the blood was collected weekly for specific antibody evaluation. At the end of the experiment, mice were anesthetized by carbon dioxide (CO_2_), euthanized by cervical dislocation, and lung and other organs were collected for further evaluation.

Firstly, SPF Balb/c mice were randomly divided into five groups (6 mice in each group) and intramuscularly immunized with indicated VLPs and adjuvant (**Table 2**, group A). Then, SARS-CoV-2 specific antibody and T-cell subset distribution were evaluated at the indicated weeks *via* Enzyme-linked immunosorbent assay (ELISA) and Flow cytometry, respectively.

**Table 2:** Information on immunization Groups.

Group		Vaccine	Dose (μg)	adjuvant	animals	Immunetimes	Interval times (weeks)	Number
A	1	S	100	MF59 (60**μ**l)+CpG (10**μ**g)	Balb/c	2	3	6
	2	SM	100	MF59 (60**μ**l)+CpG (10**μ**g)	Balb/c	2	3	6
	3	mS	100	MF59 (60**μ**l)+CpG (10**μ**g)	Balb/c	2	3	6
	4	mSM	100	MF59 (60**μ**l)+CpG (10**μ**g)	Balb/c	2	3	6
	5	PBS	–	–	Balb/c	2	3	6
B	1	mSM	100	Al (300**μ**l)+CpG (10**μ**g)	C57/BL/6NC57/BL/6N/BL/6N	1	–	8
	2	mSM	100	MF59 (60**μ**l)+CpG (10**μ**g)	C57/BL/6N	1	–	8
	3	PBS	–	–	C57/BL/6N	1	–	8
C	1	mSM	50	MF59 (60**μ**l)+CpG (10**μ**g)	C57/BL/6N	2	2	6
	2	mSM	50	MF59 (60**μ**l)+CpG (10**μ**g)	C57/BL/6N	2	3	6
	3	mSM	50	Al (150**μ**l)+CpG (10**μ**g)	C57/BL/6N	2	4	6
	4	mSM	50	Al (150**μ**l)+CpG (10**μ**g)	C57/BL/6N	2	5	6
	5	PBS	–	–	C57/BL/6N	2	–	6
D	1	mSM	50	Al (150**μ**l)+CpG (10**μ**g)	Babl/c-hACE2	2	3	6
	2	PBS	–	–	Babl/c-hACE2	2	3	6
E	1	mSM	50	Al (150**μ**l)+CpG (10**μ**g)	Balb/c	2	3	6
	2	PBS	–	–	Balb/c	2	3	6

The effects of adjuvant and duration of immune interval on antibody production were evaluated to optimize the immunization strategy. For adjuvant (**Table 2**, group B), C57/BL/6N mice were divided into three groups (8 mice in each group), and intramuscular immunization was carried out on the hind legs with the mSM and candidate adjuvants, or PBS. For immune interval (**Table 2**, group C), C57/BL/6N mice were divided into five groups (6 mice in each group), and intramuscular immunization was carried out with the mSM and candidate adjuvants or PBS for indicated intervals. In addition, SARS-CoV-2 specific antibody was evaluated at the indicated weeks *via* ELISA.

Moreover, humanized Balb/c mice were randomly divided into two groups (6 mice in each group), immunized with the mSM and Al/CpG adjuvant, or PBS, and boosted with the identical inocula at 3 weeks later (**Table 2**, Group D). SARS-CoV-2 specific antibody and neutralization activity of SARS-CoV-2 specific antibodies were determined by the pseudovirus-based neutralization assay at the indicated weeks *via* ELISA. Then, the mice were challenged with SARS-CoV-2/Wuhan-Hu-1 strain (Yan et al., 2022) (10^5.5^ TCID_50_/mL) by nasal drops (0.05 mL) 2 weeks post the boost immunization. The body weight was monitored at an interval of 2 days. Then, mice were euthanized, and viral loads in the lung were examined by real-time PCR on days 3, 5, and 7 post-infection (dpi).

To evaluate the protective efficiency of the candidate vaccine, SPF Balb/c mice were randomly divided into two groups (6 mice in each group), immunized with the mSM and Al/CpG adjuvant, or PBS, and boosted with the identical inocula at 3 weeks later (**Table 2**, group E). Then, the mice were infected with a mouse-adapted SARS-CoV-2/C57MA14 strain (Kindly provided by Prof. Yuwei Gao) (Yan et al., 2022) (10^5.5^ TCID_50_/mL) by nasal drops (0.05 mL) 10 days post the boost immunization. Mice were euthanized, and viral loads in the lung were examined by real-time PCR on 7 dpi.

### Detection of specific antibodies

SARS-CoV-2 specific antibody was examined using Mouse IgG Antibody Detection Kit for COVID-19 (ELISA) (Darui Technology, China) according to the manufacturer’s instruction. The coating antigen used in the kit was RBD protein. Briefly, serum was diluted with PBS (1:100) and added to the ELISA plate (100μL per well) at 37 °C for 40 min. After washing four times with PBS, 100μL HRP-labeled Goat pAb to Ms IgG (Darui Technology, China) or HRP-labeled Goat pAb to Ms IgG1, HRP-labeled Goat pAb to Ms IgG2a (Abcam, Britain) was added into the well and incubated at 37 °C for 20 min, followed by washing six times. Then, substrates A and B (50μL each) were added and reacted at 37 °C for 10 min in the dark. The reaction was stopped by adding 50μL stop buffer, and the absorbance values (optical density, OD) were examined at 450 nm or 630 nm using a microplate reader (TECAN SPARK, Switzerland). The serum of convalescent patients with COVID-19 and the normal person was used as the positive and negative control, respectively. Three replicates were used for each sample. When the OD value of the sample is greater than or equal to 2.1 times that of the negative control, the sample is defined as a positive. If the OD value of negative control is less than 0.1, it is calculated as 0.1.

### Neutralizing assay

The neutralization activity of SARS-CoV-2 specific antibodies was determined by the pseudovirus-based neutralization assay. Briefly, mice serum was inactivated at 56°C for 30 min, serially diluted with DMEM (3-fold dilutions), and added to a 96-well plate (50 μL/well). Then, SARS-CoV-2 pseudovirus (Du et al., 2022) was added to the well (100μL/well, MOI=100) and incubated at 37°C for 60 min. After that, Huh7 cells (2×10^5^ cells/mL) resuspended in DMEM (containing 10% FBS) were added to the well (100 μL/well) and incubated at 37°C. 48 h later, the cells were detected using One-Lumi™ Firefly Luciferase Reporter Gene Assay Kit (Beyotime, China) according to the manufacturer’s instruction and examined using a microplate reader (TECAN SPARK, Switzerland). The mock cell was used as negative control (CC), and SARS-CoV-2 pseudovirus was used as the positive control (VC). The Cut-Off Value is set as Value (sample)≤Value (VC)×0.5. The quality control is set as Value (VC)≥Value (CC)×3.

### T-cell subset distribution assay

The splenocytes were isolated from immunized mice at the end of the experiment according to the previously described protocol (Liu et al., 2013; Xu et al., 2021). Briefly, the mouse spleen was homogenized and suspended in Roswell Park Memorial Institute (RPMI) 1640 (Hyclone, Beijing, China) to prepare cell suspension. Then, the splenic lymphocytes were isolated by centrifugation using a mouse lymphocyte isolation solution according to the manufacturer’s instructions (Hao Yang Biological Manufacture Co., Ltd., Tian Jin, China). Afterward, lymphocytes were resuspended in RPMI 1640 supplemented with 10% fetal bovine serum and counted.

The splenocytes (10^6^ cells) were incubated with 1 mL PE/Cyanine7 anti-mouse CD3ϵ Antibody, FITC anti-mouse CD4 Antibody, or APC anti-mouse CD8a Antibody (Biolegend, USA) at 4 °C for 30 min, centrifuged at 1500 rpm for 5 min. Next, the pellet was resuspended in PBS and centrifuged at 1500 rpm for 5 min, followed by examination using Flow cytometry (CytoFLEX, Beckman, USA).

### Real-time PCR

Total RNA was extracted from the lung of the animals using QIAamp Viral RNA Mini Kit (QIAGEN, Germany) and examined using HiScript II U^+^ One Step qRT-PCR Probe Kit (Vazyme, China) with indicated primers (**Table 1**).

### Statistical analysis

Statistical analysis was performed using GraphPad 8.0 (GraphPad Software, SanDiego, CA) with the one-way analysis of variance (ANOVA; two-tailed, confidence intervals (CI) 95%), as indicated by the p-value. The results were statistically significant at p<0.05. At least three independent experiments were evaluated for each separate set of assays. The results are expressed as the mean ± standard deviation (SD).

## Results

### Generation of SARS-CoV-2 VLPs from the recombinant baculovirus

SARS-CoV-2 S gene was optimized and synthesized (**Figure 1A**), followed by subcloning into shuttle plasmid pFastBac™ Dual according to the protocols described previously (Xu et al., 2021), resulting in four recombinant bacmids, including pFBD-SARS-CoV-2-S, pFBD-SARS-CoV-2-SM, pFBD-SARS-CoV-2-mS, and pFBD-SARS-CoV-2-mSM (**Figure 1B**). Then, the bacmids were identified using PCR (**Supplemental Figure S1**), and the corrected bacmids were transfected into Sf9 cells to rescue recombinant baculoviruses (**Supplemental Figures S2** and **S3**), respectively. The recombinant baculoviruses are designated as rBDV-S, rBDV-SM, rBDV-mS, and rBDV-mSM. Thereafter, the expression of exogenous genes in the Sf9 cells was evaluated by IFA. As shown in **Figure 1C**, the cells infected by recombinant baculoviruses exhibited apparent fluorescence compared with the mock-infected group, suggesting exogenous genes were efficiently expressed in the cells. These results were further confirmed by Western blot. As expected, four kinds of S proteins can be detected in cells infected with the recombinant baculovirus (**Figure 1D**). There were several bands in the wild-type groups, while the optimized S protein (mS) had only one specific band at 180 kDa, indicating the optimized protein is more stable and not easily degraded by protease than the wild-type S. Moreover, the M1 protein of H5N1 can be detected between 22 and 25 kDa (**Figure 1D**), which further proves the successful expression of these proteins.

To prepare VLPs, sf9 cells were infected with recombinant baculoviruses, and the protein was purified by ultracentrifugation with sucrose cushions, followed by transmission electron microscope observation (TEM). As shown in **Figure 1E**, the VLPs showed spherical shapes as typical SARS-CoV-2 virions. Spike structure can be observed around VLP, similar to the characteristic spike structure of intact virus particles, indicating that the recombinant SARS-CoV-2 VLPs were generated successfully. The expression of *mSM* genes was more effective than in the other three groups, suggesting that codon optimization can enhance the expression of exogenous genes in the Sf9 cells.

### SARS-CoV-2 VLPs induced effective immune responses in mice

**Figure 2:**
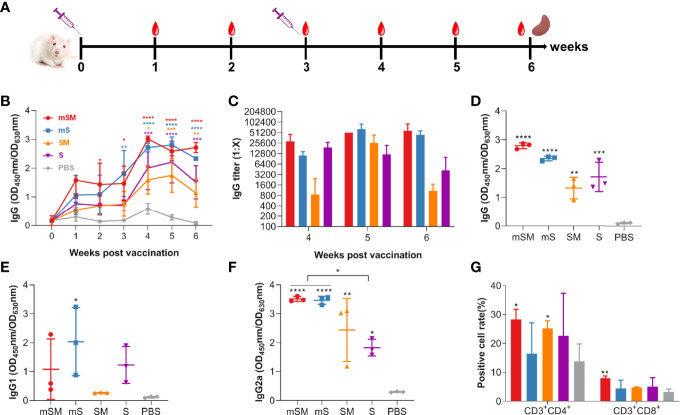
Evaluation of the immunogenicity of the VLP. Mice were primed with PBS or SARS-CoV-2 VLPs and boosted three weeks after the prime immunization using the same inocula used for priming. Blood samples were collected from the tail vein of mice at indicated times after the initial vaccination and used to analyze humoral immune responses using an antibody detection kit (The coating antigen used in the kit was RBD protein). Sera collected from the tail vein of pre-immune mice were used as a negative control. *, p< 0.05; **, p< 0.01; ***, p< 0.001; ****, p< 0.0001. The results are expressed as the mean ± standard deviation (SD). **(A)** Schematic diagram of immunization. **(B)** changes of total IgG during the immunization. **(C)** IgG titers at 4, 5, and 6 weeks post initial immunization. **(D-F)** RBD-specific IgG **(D)**, IgG1 **(E)**, and IgG2a **(F)** at six weeks post initial immunization. **(G)** Splenic lymphocyte subtypes.

Mice were immunized with the purified VLP or PBS, followed by an evaluation of the immune responses. Animals were monitored daily for adverse effects. No obvious adverse events were observed. Blood was collected at 0, 1, 2, 3, 4, 5, and 6 weeks after the first dose of the immunization, and the boost was conducted three weeks after the first vaccination (**Figure 2A** and **Table 2**, group A).

As shown in **Figure 2B**, levels of SARS-CoV-2 specific antibody increased gradually in the VLP groups after the first dose of the immunization, which was significantly higher than that of the PBS group. The highest titer of specific antibodies in serum reached the peak four weeks post the first immunization in the VLP groups, with the highest level in the mSM group, and then decreased slightly in each group. After the boost immunization, the titers of the mSM and mS groups were higher than those of other groups, up to 1: 102,400 (**Figure 2C**). Compared with the control group, IgG, IgG1, and IgG2a at six weeks post initial immunization in the immunized groups increased to different degrees (**Figures 2D–F**), and the levels of specific IgG2a antibodies were higher than that of IgG1 (**Figures 2E, F**). In addition, the levels of specific IgG2a in the mSM and mS groups were higher than in other infected groups (**Figure 2F**). Three weeks post the boost immunization, the splenocytes were isolated from immunized mice according to a previously described protocol (Liu et al., 2013), followed by an evaluation of the T-cell subset distribution. As shown in **Figure 2G**, the levels of CD3^+^CD4^+^T and CD3^+^CD8^+^T lymphocytes in the mSM group were highest than in the other groups. These results suggest that the optimized S proteins, especially the mSM protein, had more robust immunogenicity than the wild-type S. Therefore, recombinant baculovirus rBDV-mSM and the optimized mSM were further evaluated in the subsequent studies. Then, the mSM protein in the supernatant was further purified using a stepwise sucrose density gradient and evaluated using SDS-PAGE, Western blot, and ELISA. Figure The purified mSM protein can be efficiently recognized by the convalescent serum of a COVID-19 patient (primary antibody), while the unpurified protein in the supernatant has no obvious band (**Supplemental Figure S4**). The concentration of the purified mSM protein was quantified *via* standard curve (**Supplemental Figure S5**), and it was 0.2 μg/μL.

**Figure 3:**
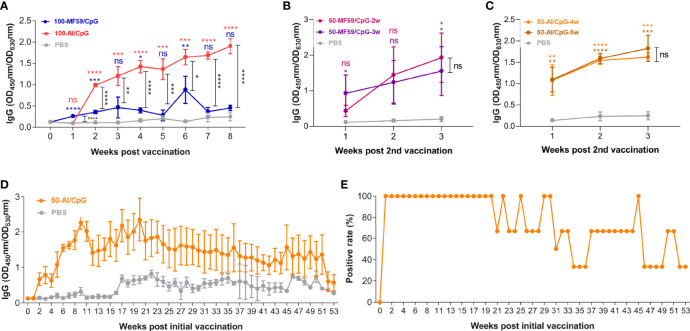
Optimization of immunization strategy. **(A)** Adjuvant. **(B, C)** Immune interval duration. **(D)** RBD-specific IgG. **(E)** Proportion of positive serum of immunized mice in Figure3D (positive rate). Differences between vaccine groups are indicated by black asterisks. Differences between vaccine group and PBS are indicated by color asterisks.

To optimize the immunization strategy of the mSM group, we evaluated the effects of adjuvant and duration of immune interval on antibody production and compared it with that of the PBS group (**Table 2**, group B-C). As shown in **Figure 3A**, the effect of the Al/CpG adjuvant on antibody production was better than MF59/CpG adjuvant. However, the duration of the immune interval has no significant effect on antibody production (**Figures 3B** **, C**). Meanwhile, the optimized immunization with mSM and Al/CpG adjuvant can stimulate animals to produce sustained high-level antibodies, which can still be detected after 53 weeks of vaccination (**Figure 3D**). The proportion of positive serum of immunized mice in **Figure 3D** (positive rate) was about 33.33% (**Figure 3E**).

### SARS-CoV-2 VLPs induced effective immune responses in humanized mice

**Figure 4:**
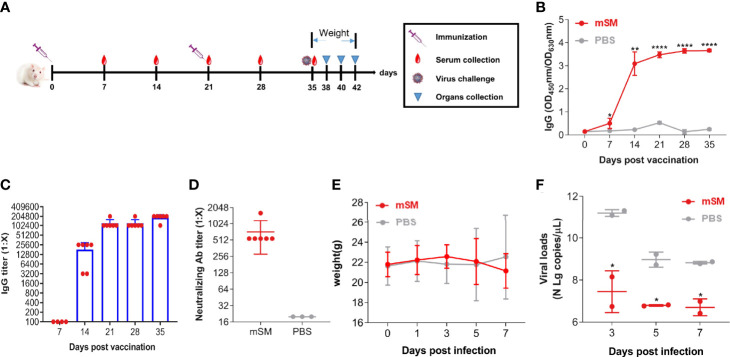
Evaluation of the immune effect of the VLP in humanized mice. Humanized mice were primed with PBS or SARS-CoV-2 VLP and boosted three weeks after the prime immunization using the same inocula used for priming. Blood samples were collected from the tail vein of mice at indicated times after the initial vaccination and used to analyze humoral immune responses using an antibody detection kit. Sera collected from the tail vein of pre-immune mice were used as a negative control. *, P< 0.05; **, P< 0.01; ****, P< 0.0001. The results are expressed as the mean ± standard deviation (SD). **(A)** Schematic diagram of immunization. **(B)** RBD-specific IgG. **(C)** IgG titer. **(D)** Neutralizing antibody. **(E)** Bodyweight. **(F)** Virus loads detected by qRT-PCR.

To clarify the immunogenicity and protective efficiency of the VLP-based vaccine, humanized ACE2 mice (Balb/c-hACE2) were immunized with the VLP or PBS, followed by an evaluation of the immune responses and virus challenge (**Figure 4A** and **Table 2**, group D). **Figures 4B** **, C** shows that the optimized vaccinaion with mSM and Al/CpG adjuvant can stimulate humanized mice to produce sustained high-level antibodies at 14, 21, 28, and 35 days post initial vaccination, with the highest titer of about 1:204,800 at 35 days post initial immunization. Furthermore, the titers of neutralizing antibodies reached 1:1,620 on the 35^th^ day of the initial vaccination (**Figure 4D**).

Furthermore, the immunized mice were infected with SARS-CoV-2/Wuhan-Hu-1 14 days post the boost immunization, and animals were monitored at an interval of 2 days for adverse effects. As a result, no apparent adverse events were observed, and the body weights of the vaccine-immunized group were similar to that of the PBS group (**Figure 4E**). Moreover, the viral loads of the vaccine-immunized group were significantly decreased compared with that of the PBS group 3 days post-challenge (**Figure 4F**). These results indicate that the optimized immunization with SARS-CoV-2 VLP (mSM) and Al/CpG adjuvant can induce effective immune responses.

### SARS-CoV-2 VLPs can efficiently protect the animal against virus challenge

**Figure 5:**
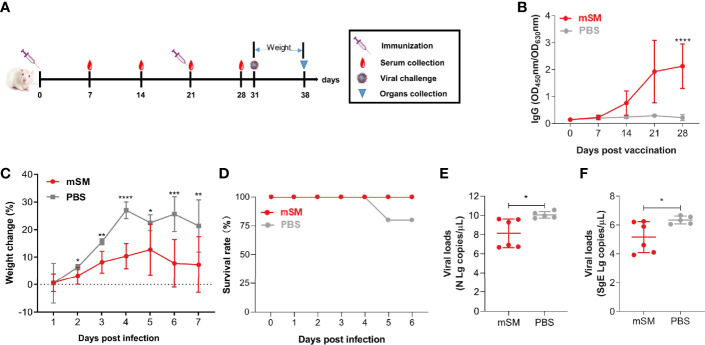
The mSM-based vaccine is effective against virus challenges. Mice were primed with PBS or SARS-CoV-2 VLP and boosted three weeks after the prime immunization, followed by virus challenge at 31 days post-prime immunization. Blood samples were collected from the tail vein of mice at indicated times after the initial vaccination and used to analyze humoral immune responses using an antibody detection kit. Sera collected from the tail vein of pre-immune mice were used as a negative control. *, P< 0.05; **, P< 0.01; ***, P< 0.001; ****, P< 0.0001. The results are expressed as the mean ± standard deviation (SD). **(A)** Schematic diagram of immunization and infection. **(B)** RBD-specific IgG. **(C)** Bodyweight. **(D)** Animal survival. **(E, F)** Virus loads detected by qRT-PCR.

To further clarify the protective efficiency of the mSM-based vaccine, BABL/c mice were immunized with the VLP or PBS, followed by an evaluation of the immune responses and challenge with a mouse-adapted SARS-CoV-2 strain (**Figure 5A** and **Table 2**, group E). As shown in **Figure 5B**, the SARS-CoV-2 specific antibody increased gradually in the mSM group, but there was no significant increase in the PBS group. There was a significant difference between the two groups 28 dpi. Furthermore, the body weights of the two groups decreased gradually after being challenged with the virus at 10 days post the boost immunization, but the weight-loss trend of the mSM-immunized mice was significantly lower than that of the control group (**Figure 5C**). On the fifth day of the virus challenge, one mouse in the PBS group died, while the others survived. At the end of the experiment, the survival rate of the mSM group was 100%, and that of the PBS group was 83.3% (**Figure 5D**). Then, the viral loads were examined on the seven^th^ dpi. As shown in **Figure 5E** and 5F, the copy numbers of viral genomic RNA (N gRNA, **Figure 5E**) and viral subgenomic RNA (sgERNA, **Figure 5F**) in the lung of the mSM group decreased significantly from that of the PBS group. These results indicate that the candidate vaccine based on the mSM VLP and Al/CpG adjuvant can establish an effective protective barrier to protect mice from virus attacks.

## Discussion

The Spike of β-coronavirus is the main target of vaccine and therapeutic drug development (Du et al., 2009). Most vaccines of SARS-CoV-2, including subunit vaccines, mRNA vaccines, DNA vaccines, and viral vector vaccines, were constructed based on the Spike or the spike S1 or RBD (RPM) domain (Gao et al., 2020; Smith et al., 2020; Wang et al., 2020a; Zhu et al., 2020; Flanagan et al., 2021; Li et al., 2021b). A previous study indicated that the chimeric VLPs containing the SARS-CoV-1 Spike (with a replacement of the TM/CT by the corresponding sequence of influenza virus HA) and the influenza M1 could be highly expressed in the baculovirus insect cell expression system (Liu et al., 2011). Furthermore, inserting two prolines in the S2 subunit of MERS-CoV and SARS-CoV can effectively stabilize the prefusion conformation of the Spike and improve its stability, which also has been applied to SARS-CoV-2 vaccine development (Kirchdoerfer et al., 2018; Dagotto et al., 2020; Wrapp et al., 2020). Moreover, the mutation in the furin cleavage site (RRAR) between 682-685 in the S1 subunit of the MERS-CoV spike could enhance the homogeneity and stability of the MERS-CoV vaccine (Kirchdoerfer et al., 2018; Dagotto et al., 2020; Wrapp et al., 2020; Kalnin et al., 2021). Moreover, ADE has been reported in several viruses *in vitro* or *in vivo*, such as West Nile fever virus (WNV), Dengue virus (DENV), Ebola virus (EBOV), as well as in coronavirus infections, feline infectious peritonitis virus (FIPV), and SARS-CoV-1 (Negro, 2020). Therefore, although there has no clear evidence that ADE plays a role in the pathogenesis of COVID-19, it is crucial to avoid ADE in vaccine development. Besides, it was reported that a linear epitope (S597-603) of the SARS-CoV-1 spike protein could enhance virus infection *in vitroin vitro* and in non-human primates (Wang et al., 2016). Therefore, due to the above consideration, we constructed a chimeric VLP of SARS-CoV-2 spike protein and H5N1 M1 based on the baculovirus and insect cell expression system. The results showed that the Spike and H5N1 M1 were expressed successfully, and chimeric VLPs could be formed (**Figure 1**). The protocol for preparing a large number of high-purity VLPs is being optimized in our lab.

Notably, the expression of the SARS-CoV-2 Spike and the formation of VLPs are different in different expression systems. SARS-CoV-2 vaccines based on VLPs have been reported (Ghorbani et al., 2020; Biswas et al., 2021; Chu et al., 2021a; Geng et al., 2021; Ward et al., 2021; Boix-Besora et al., 2022; Hemmati et al., 2022; Mohsen et al., 2022), some expressed by mammalian cells or plant cells, while others are based on the RBD. However, only the immunogenicity of most of these VLPs has been studied, and there is no report on the preparation of SARS-CoV-2 VLP by insect baculovirus system. Meanwhile, it was reported that the plant-derived VLP vaccine for COVID-19 was immunogenic, which can be significantly enhanced by adjuvant (Ward et al., 2021), suggesting that the vaccine based on VLPs is promising for the control and prevention of the COVID-19 pandemic. Previous studies also found that optimizing codon usage preference can enhance the expression and formation of VLPs (Wang et al., 2020b; Xu et al., 2021). As reported, three sites of the Spike, including D614, RRAR (682-685), and KV986-987, are crucial for the virus infection. The mutation of D614 to A614 may avoid Antibody-dependent enhancement (ADE) (Wang et al., 2016). The mutation of RRAR (682-685) to GSAS can inhibit the recognition and cleavage of furin (Kalnin et al., 2021). The mutation of KV986-987 to PP986-987 can enhance immunogenicity (Kirchdoerfer et al., 2018; Dagotto et al., 2020; Wrapp et al., 2020). Therefore, these sites were replaced by the corresponding sequence, respectively, and the optimized sequence was designated the *mS* gene (**Figure 1A**). Compared with the unoptimized VLPs, the optimized S protein was more stable and not easily degraded by protease than the wild-type S (Fig1), and it is easier to form VLPs (Fig1). Vaccines based on the codon-optimized VLP can induce higher levels of specific IgG antibodies than the wild-type S. Furthermore, the optimized S protein chimeric with H5N1 M1 (the mSM group) showed better immunogenicity than other groups. It could induce higher levels of specific IgG antibodies and cellular immune responses. Further studies proved that VLPs based on the optimized S protein (the mSM group) can induce immune responses in mice more effectively than wild-type S, provide protection for mice after challenge, reduce virus load and improve survival rate. The viral load in mice decreased obviously, and the survival rate of mice increased obviously.

Moreover, the adjuvant and duration of immune interval of the mSM-VLP-based vaccine were also optimized, followed by evaluation in normal and humanized mice. As expected, the mSM-VLP-based vaccine adjuvanted with Al/CpG can induce high levels of specific IgG antibodies, and the highest titer of neutralizing antibody is 1: 1620. The vaccine can reduce weight loss, significantly reduce the viral load in the lungs of mice, and have an apparent protective effect against virus attack. However, the duration of the immune interval has no significant effect on antibody production (**Figures 3B** **, C**). Meanwhile, the optimized immunization with mSM and Al/CpG adjuvant can stimulate animals to produce sustained high-level antibodies, lasting at least 53 weeks post the initial vaccination, with a positive rate of about 33.33% (**Figures 3D** **, E**). These results provide theoretical guidance for designing high-efficiency vaccines, optimizing immunization programs, and developing vaccines against SARS-CoV-2 variants. The construction and research of VLPs for Delta and Omicron variants are in progress.

## Conclusion

In conclusion, chimeric VLPs based on the optimized Spike of SARS-CoV-2 and H5N1 M1 protein were constructed using the baculovirus system. The chimeric VLP adjuvanted with Al/CpG can induce effective and sustained immune responses against virus challenges in mice. The present study results provide theoretical guidance for designing high-efficiency vaccines, optimizing immunization programs, and developing vaccines against SARS-CoV-2 variants.

## Data availability statement

The original contributions presented in the study are included in the article/**Supplementary Material**. Further inquiries can be directed to the corresponding author.

## Ethics statement

The animal study was reviewed and approved by the experimental animal committee of Laboratory Animal Center, Changchun Institute of Veterinary Medicine, Chinese Academy of Agricultural Sciences.

## Author contributions

Conceptualization, LR, CL, and NJ; methodology, WX and JC; validation, LL, LY, and YJ; formal analysis, PH and ZX; data curation, WZ and JC; writing—original draft preparation, JC and LR; writing—review and editing, LR and CL; visualization, PL, MT and ZG; supervision, CL and NJ; project administration, CL and NJ; funding acquisition, CL and NJ. All authors have read and agreed to the published version of the manuscript.

## Funding

This work was supported by the National Key Research and Development Program of China [No. 2021YFD1801103-6]; the National Natural Science Foundation of China [No. 31972719]; CAMS Innovation Fund for Medical Sciences [2020-12M-5-001]. The funders had no role in study design, data collection and analysis, publishing decisions, or manuscript preparation.

## Conflict of interest

The authors declare that the research was conducted in the absence of any commercial or financial relationships that could be construed as a potential conflict of interest.

## Publisher’s note

All claims expressed in this article are solely those of the authors and do not necessarily represent those of their affiliated organizations, or those of the publisher, the editors and the reviewers. Any product that may be evaluated in this article, or claim that may be made by its manufacturer, is not guaranteed or endorsed by the publisher.
