# Supplementary material for: Immunogenicity and protective potential of chimeric virus-like particles containing SARS-CoV-2 spike and H5N1 matrix 1 proteins
Source: Front Cell Infect Microbiol. 2022 Jul 18;12:967493. doi: 10.3389/fcimb.2022.967493 (PMC9339902; doi:10.3389/fcimb.2022.967493)
Supplement: Supplementary file 1 [file DataSheet_1.docx]

**Supplemental figures**

**Supplemental Fig. S1: PCR identification of the bacmids.**


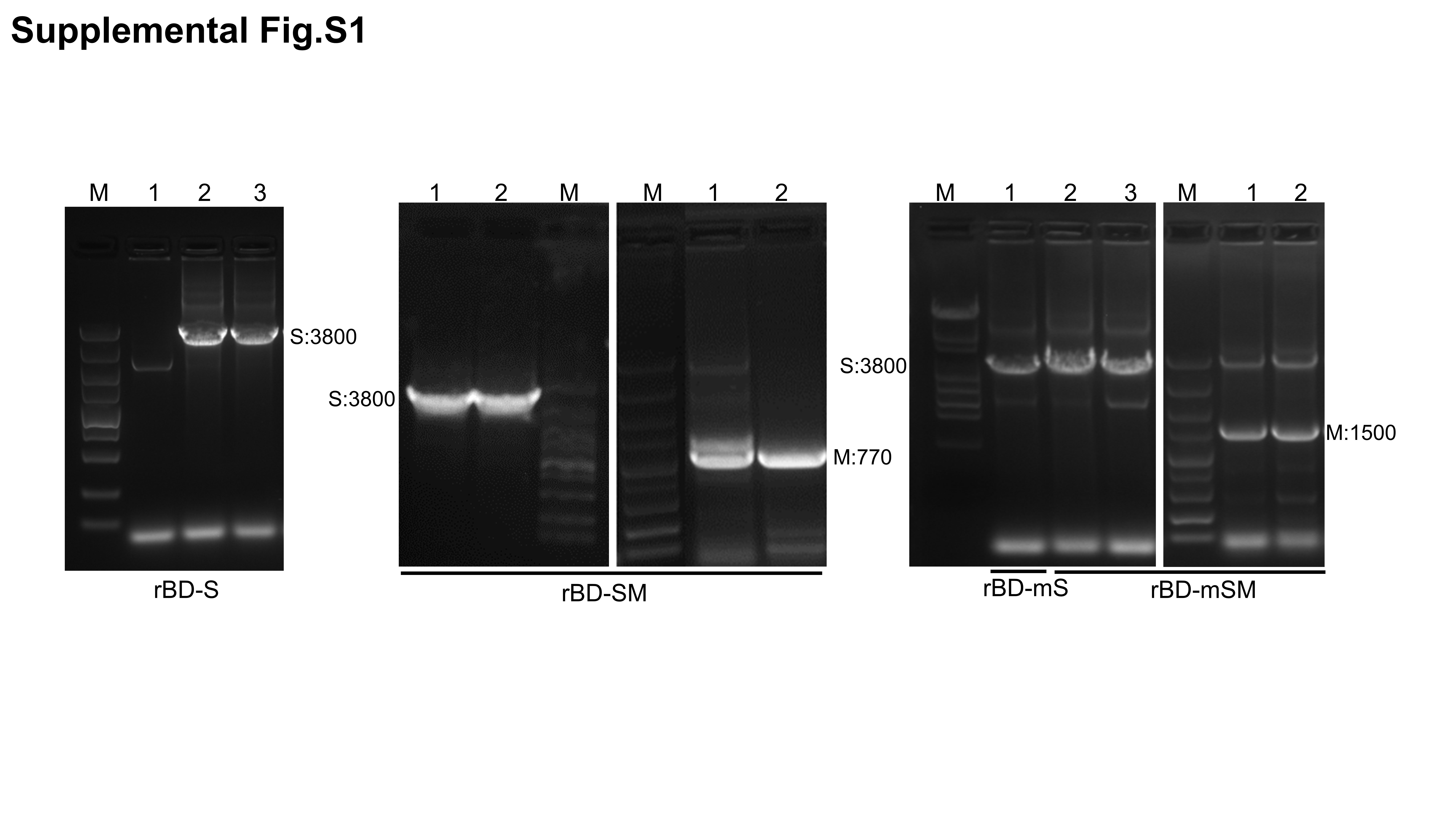


**Supplemental Fig. S2: Schematic diagram of recombinant baculoviruses rescue.**


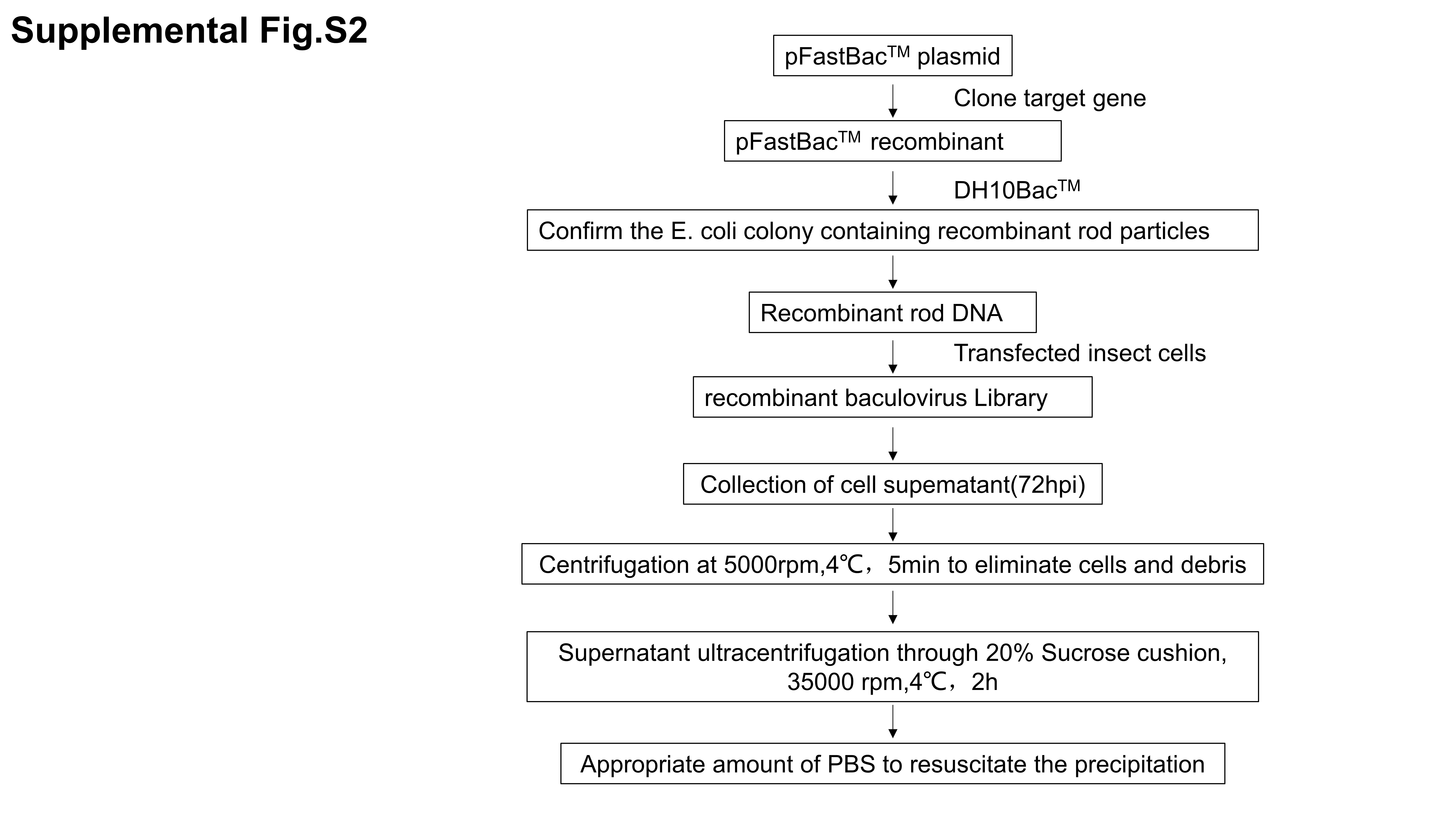


**Supplemental Fig. S3: recombinant baculoviruses**


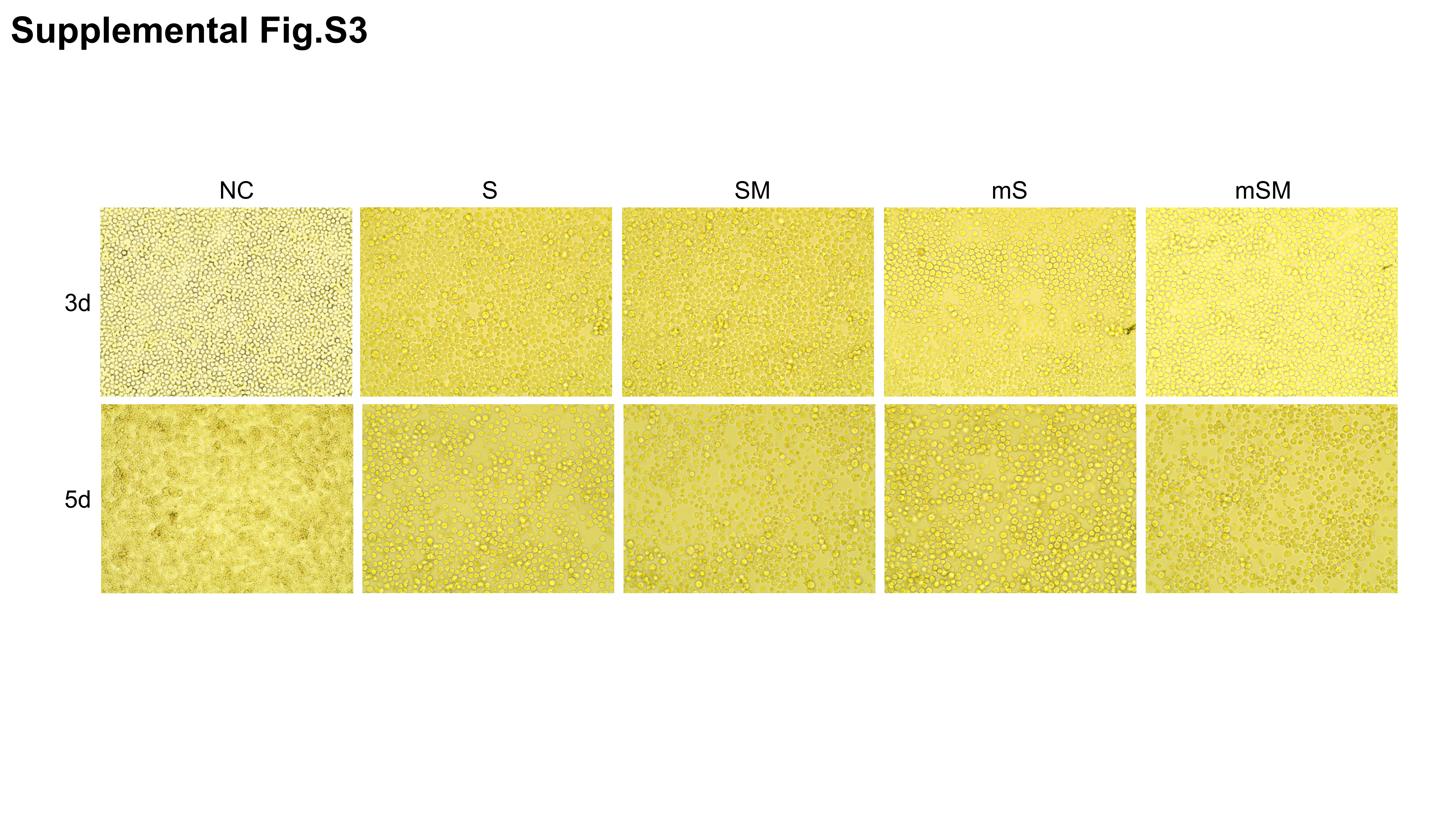


**Supplemental Fig. S4:** Purification of mSM. Convalescent serum of COVID-19 patient as the primary antibody, and HRP-labeled Goat Anti-Mouse IgG (H+L) as the secondary antibody. M, protein marker; Before, before purification; After, after purification.

kDa

Befor

After

M

mSM


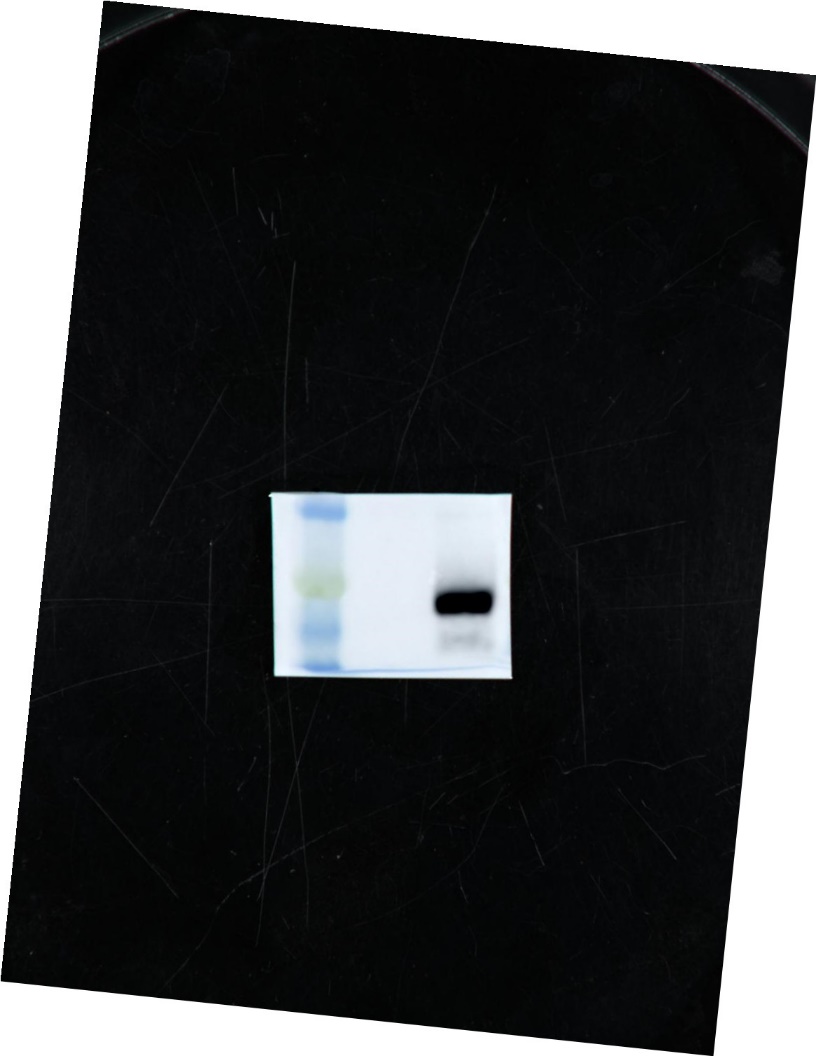

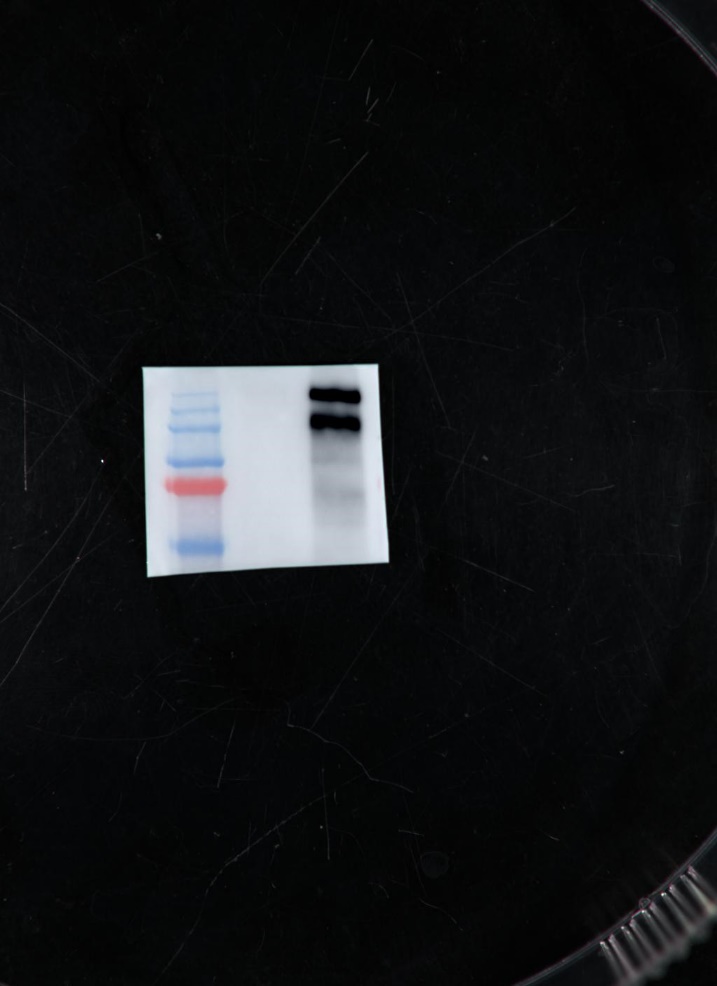

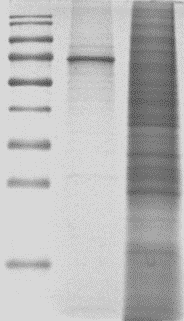


Befor

After

mSM

25

240

180

**Supplemental Fig. S5:** Standard curve.

**Supplemental Fig. S6: Original Western images used for preparing Figure.1D.**

| rBV-S S | 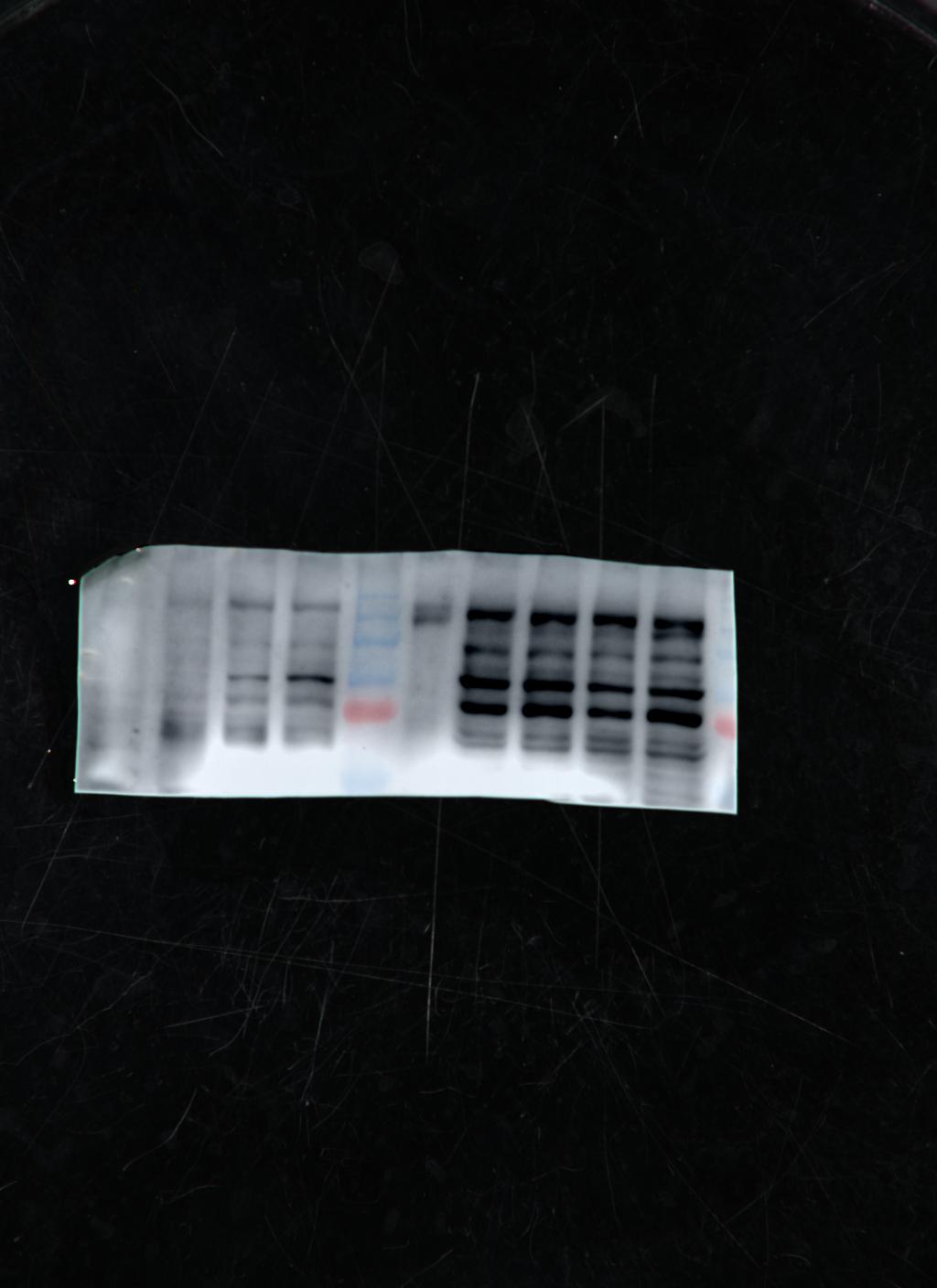 |
| --- | --- |
| rBV-SM S | 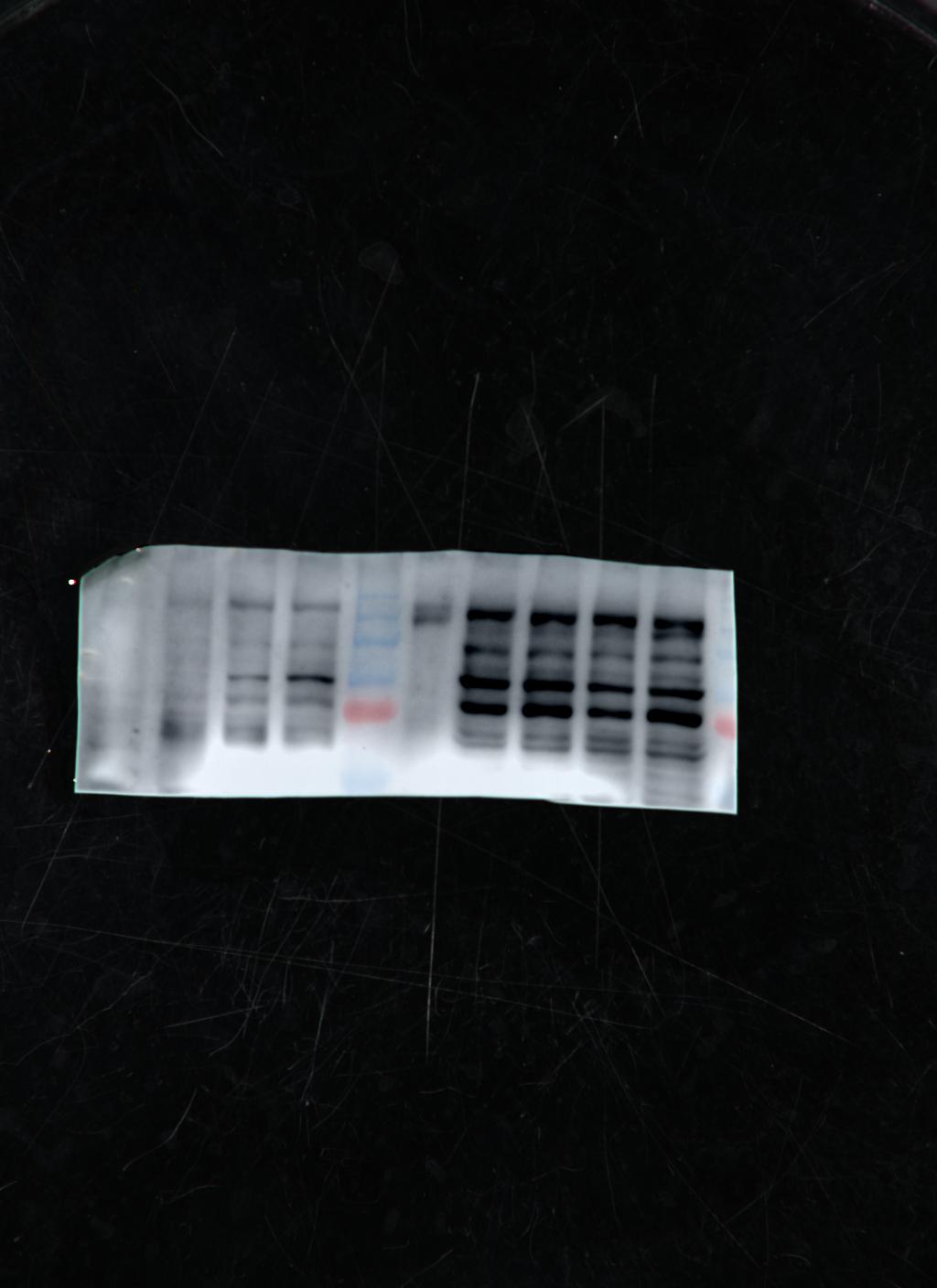 |
| rBV-SM M1 | 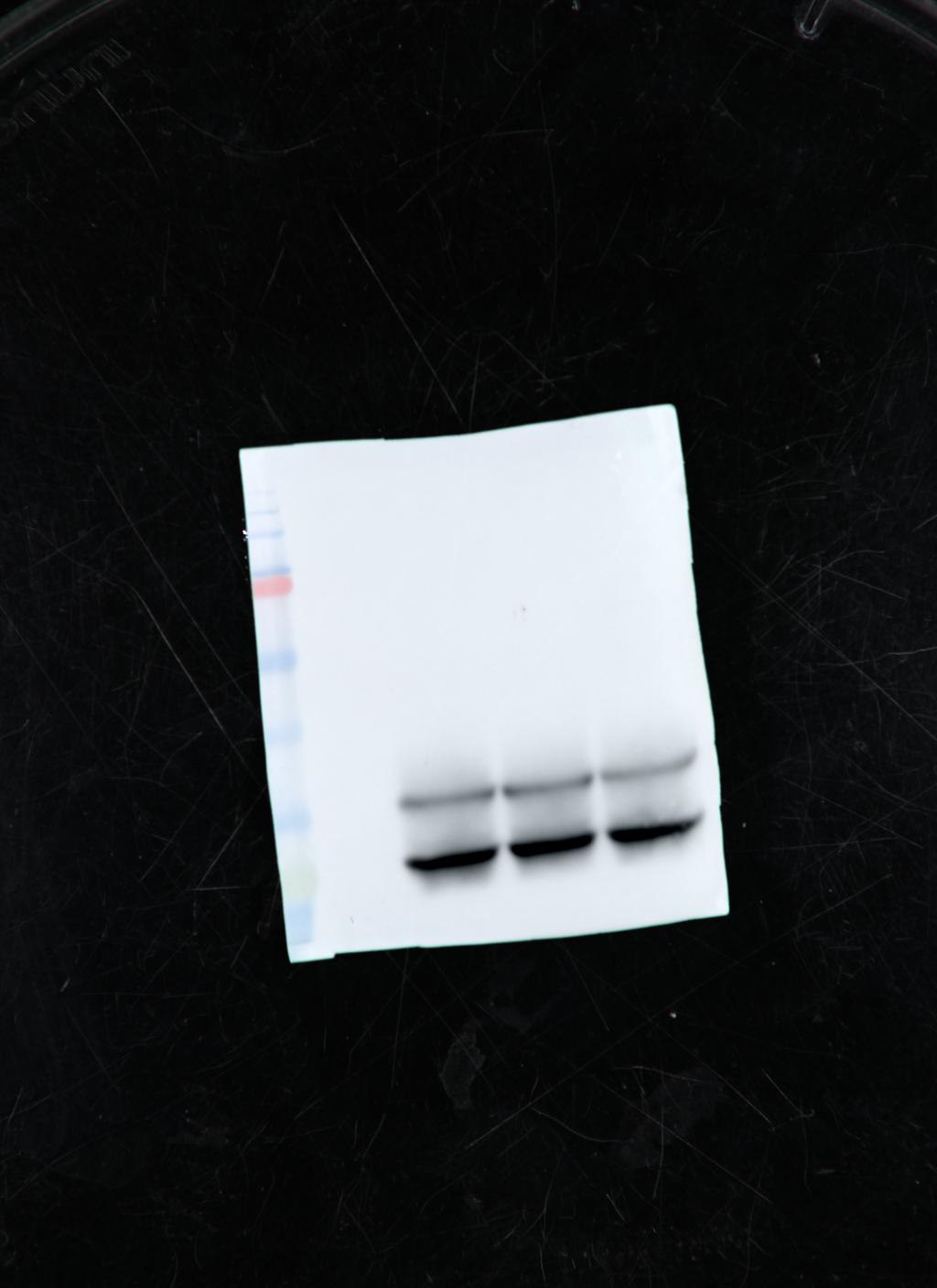 |
| rBV-mS mS | **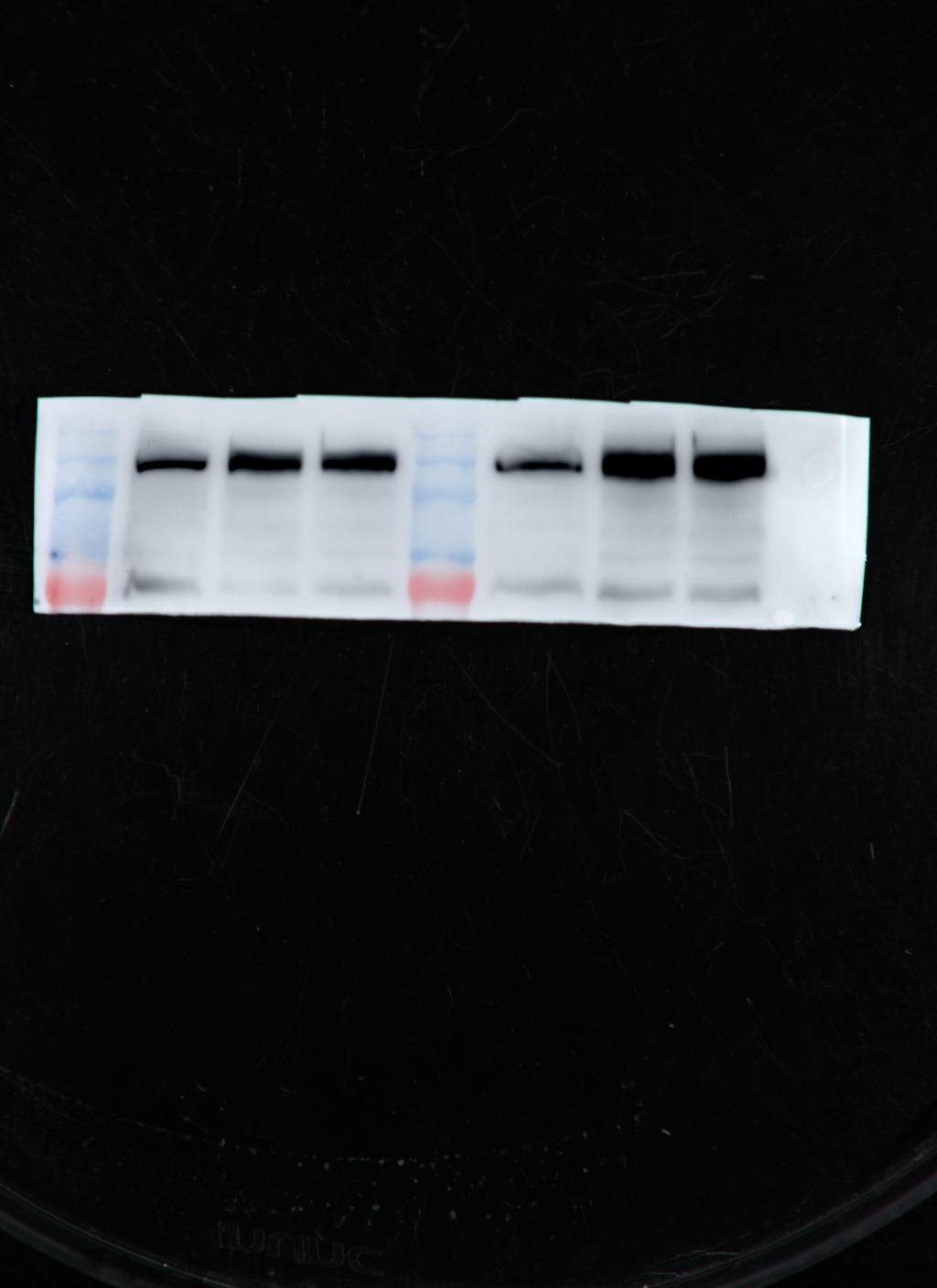** |
| rBV-mSM mS | 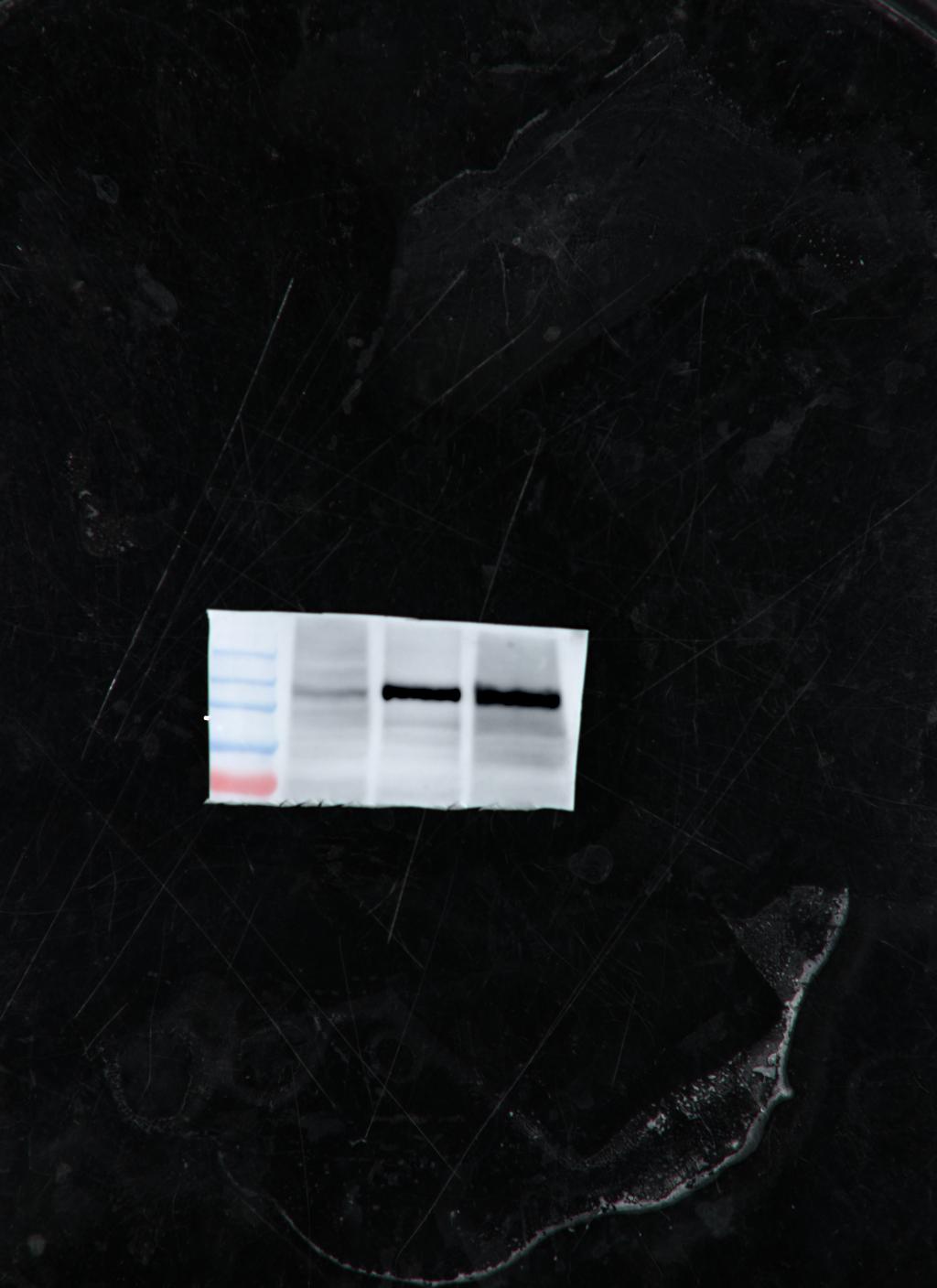 |
| rBV-mSM M1 | 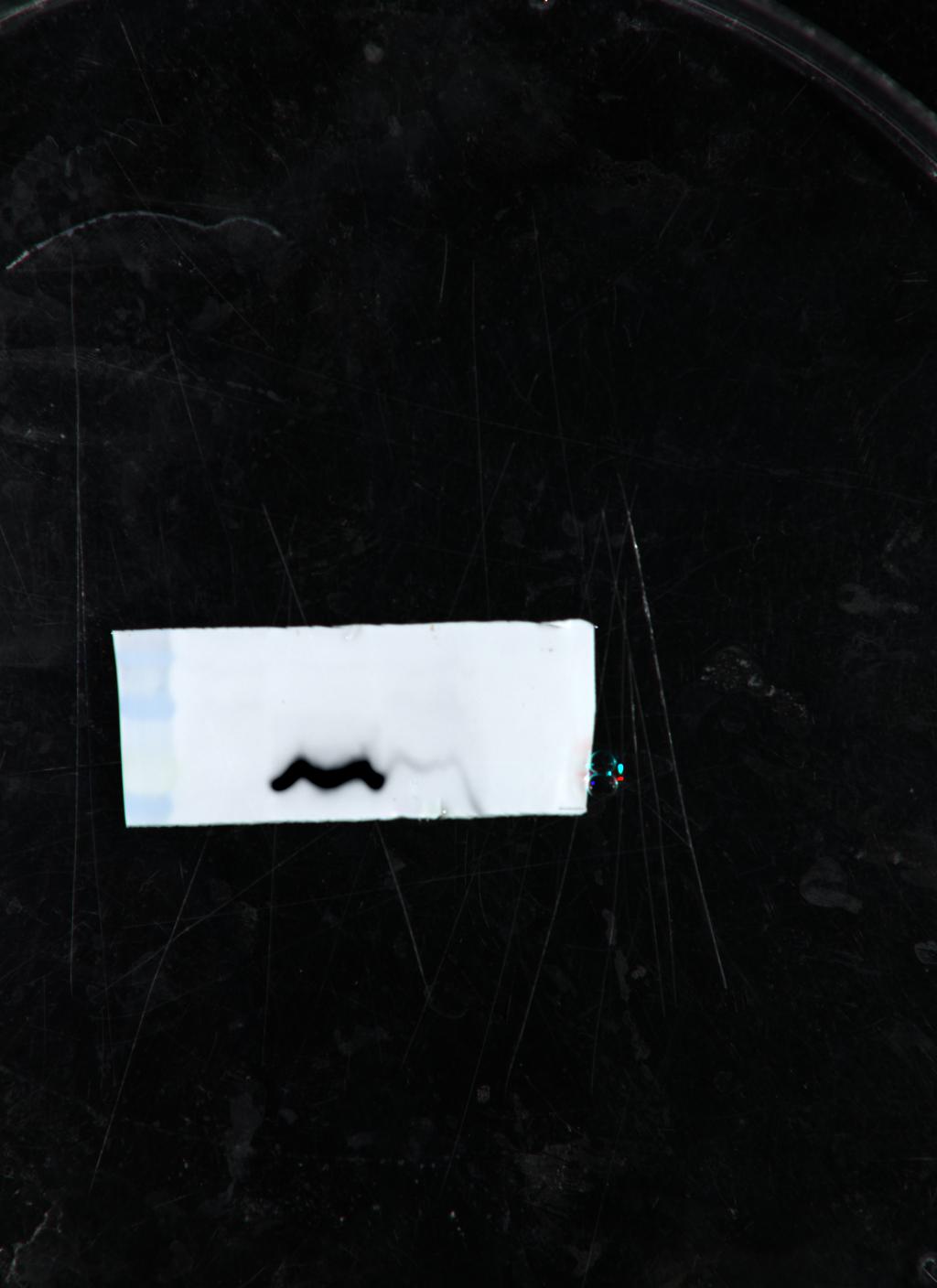 |
